# Supplementary material for: Capacity Coaching: A Focused Ethnographic Evaluation in Clinical Practice
Source: Mayo Clin Proc Innov Qual Outcomes. 2020 Feb 17;4(2):190–202. doi: 10.1016/j.mayocpiqo.2019.11.002 (PMC7140014; doi:10.1016/j.mayocpiqo.2019.11.002)
Supplement: Supplemental Appendix 1 [file mmc1.docx]

**Capacity Coaching: A Focused Ethnographic Evaluation in Clinical Practice**

Kasey R. Boehmer, MPH, PhD^1^

Anjali Thota, BA^1^

Paige Organick, BA^1^

Kathryn Havens, MD^2,3^

Nilay D. Shah, PhD^4^

1. Knowledge and Evaluation Research (KER) Unit, Mayo Clinic, Rochester, MN, USA
2. Kern Institute, Medical College of Wisconsin
3. Women’s Health Clinic, Zablocki VA (appointment at time of pilot)
4. Health Sciences Research, Mayo Clinic, Rochester, MN, USA

**Corresponding Author:**

Kasey R. Boehmer, PhD, MPH

200 First Street SW

Rochester, MN 55901

[Boehmer.kasey@mayo.edu](mailto:Boehmer.kasey@mayo.edu)

Phone: 507-293-0175

**Financial Support:** The data collection and analyses for this manuscript was supported by R03HS 25517. Publication was supported by U54TR 01621. Carry out of the Capacity Coaching program at the VA Medical Centers was supported by VA Innovators Network Funding Cycle 2016-2017.

**Conflict of Interest:** The authors have no relevant conflicts of interest to report.

**Key Words:** Coaching, Health and Wellness Coaching, Capacity, Capacity Coaching, Minimally Disruptive Medicine

**Wordcount: 3,000**

**Abstract**

**Objective:** To qualitatively evaluate the implementation of *Capacity Coaching*, an intervention to address the work patients must undertake to manage their conditions, implemented as a quality improvement pilot in one of two implementing Veterans’ Administration (VA) Medical Centers.

**Patients and Methods:** Two VA Medical Centers in the Midwest sought to implement *Capacity Coaching* as a quality improvement pilot in their Patient-Aligned Care Teams (PACT) for six months (April 1, 2017 - October 31, 2017). Following the pilot, we conducted a focused ethnography (on-site data collection January 2 – 4, 2018), including interviews, a focus group, and observations with staff at one site to assess the implementation of *Capacity Coaching*. Data were analyzed inductively and findings were cross-referenced with implementation theory.

**Results:** We found that implementation was feasible and achieved changes that were aligned with reducing patient work and increasing capacity. We found that the key facilitators for the implementation of this program were in participants making sense of the intervention (coherence) and working collectively to enact the program (collective action). Whereas, the main challenges for the program were in planning the work of implementation and enrolling a diverse coalition of staff to expand referrals to the program (cognitive participation), and in evaluating the impact of the program on outcomes that upper leadership was interested in (reflexive monitoring).

**Conclusion:** Implementation of *Capacity Coaching* is feasible in clinical practice and may be a promising intervention for the care of chronic conditions. Further research should focus on testing *Capacity Coaching* using these lessons-learned.

**Abbreviations**

BREWS – Biography, Resources, Environment, Work, Social (pneumonic, Theory of Patient Capacity)

CuCoM – Cumulative Complexity Model

HbA1c – Hemoglobin A1c

ICAN – ICAN Discussion Aid

MDM – Minimally Disruptive Medicine

LSL – Leadership Saves Lives

NPT – Normalization Process Theory

PACT – Patient-Aligned Care Team

VA – Veteran’s Administration

**Background**

Epidemiologic studies show that by middle-age, one-half of the population already lives with one chronic condition and one-third already have two or more chronic conditions.^1^ Once a problem of the elderly, multimorbidity is now a reality for many working-age adults. As more treatments and tests are added to the regimen for these patients to manage their conditions, an important problem surfaces – the competing priorities of life. Patients with multimorbidity are at increased risk for “structural non-compliance,” which is the inability to adhere to care plans because the number and complexity of the healthcare tasks are simply overwhelming.^2^ May and colleagues, proposed a framework to address this challenge and called it Minimally Disruptive Medicine (MDM).^2^

MDM is a model of care that is oriented by an overarching conceptual model: the Cumulative Complexity Model (CuCoM).^3^ The CuCoM illustrates that there is patient *work* necessary to implement health interventions (e.g. attending appointments, taking medications, self-monitoring).^3-5^ Second, patients invest *capacity* to implement patient work, which is the same capacity that they must use for life activities (e.g. care for families, employment).^3,6^ When patient work exceeds capacity, this impairs patients’ abilities to access and use healthcare and enact self-care, and may result in worsening health outcomes.^3^ Two middle-range theories describe the concepts of workload and capacity used in the CuCoM in greater detail: the Normalization Process Theory (NPT) and the Theory of Patient Capacity (TPC), respectfully.^4,5,7,8^

Briefly, NPT focuses on four key domains: *coherence*, how participants make sense of the work required to take up the intervention; *cognitive participation* to enroll others and plan the work; *collective action* to enact the work of implementing the intervention; and *reflexive monitoring*, to appraise if the intervention is worth the effort. ^4,5,8^ NPT can be used at the individual level to understand patient behavior and at the team level to understand healthcare teams’ implementation of interventions. The TPC describes patient capacity as a dynamic interaction between patients and their **B**iographies, **R**esources, **E**nvironment, patient **W**ork, and **S**ocial network.^7^ **Figure 1** demonstrates the relationships between MDM’s conceptual models and theories.

*Capacity Coaching* is a patient-centered intervention that has been designed in alignment with MDM. It is novel in its application of MDM, but also its combination of two types of interventions that have been used separately for populations with multimorbidity – intensive team-based management and coaching.^9^ *Capacity Coaching* begins the first coach-patient conversation with the ICAN Discussion Aid (**Appendix 1**), which was designed to support MDM in practice to understand patients healthcare workload and capacity.^10^ *Capacity Coaching* builds upon traditional Health and Wellness Coaching (HWC), but is specifically designed to work within the context of the healthcare team to reduce treatment burden and increase capacity for overwhelmed patients living with chronic illness.^9^

*Aim*

To date, *Capacity Coaching* remains untested. It is unknown whether it can be implemented into primary care, nor if implementation is successful, how it would impact patient outcomes. The purpose of this study was to understand its feasibility for implementation in practice. Understanding if this intervention is feasible to implement clinically, as well as key barriers and facilitators to its implementation, can enable the testing of Capacity Coaching in a larger clinical trial.

**Methods**

Two Veterans’ Administration (VA) Medical Centers in the Midwest sought to implement *Capacity Coaching* as a quality improvement pilot initiative in their Patient-Aligned Care Teams (PACT). In one location, four PACT teams in the Women’s Health Clinic implemented the program (Site 1); at the other location, the mental health care PACT team in the Women’s Health Clinic implemented the program (Site 2). PACT teams include physicians, nurses, social workers, pharmacists, nutritionists, and peer-support specialists (PSS). The teams were trained during a one-day workshop delivered by K.B.; approximately 40 team members across the five PACT teams were trained. Those trained included all members from Site 1 and two team members from Site 2. The workshop content was delivered over a single-day 8-hour session. It included training on MDM and its conceptual and theoretical models, the ICAN Discussion Aid, Capacity Coaching principles, and implementation in complex systems. It also included facilitated planning sessions for conceptualizing Capacity Coaching implementation in practice.

Following the workshop, sites independently planned and implemented the program in their settings; K.B. provided limited remote support. Following the closure of the implementation period, we returned to Site 1 to understand the program’s implementation successes and challenges. While returning to both clinics would have been ideal, due to pragmatic time and funding constraints, we sought to collect data from Site 1, as it was where the workshop occurred and housed four of the five PACT teams trained. We used focused ethnographic methods to accomplish our aims. Focused ethnography is similar in its methods to traditional ethnography, but instead focuses on answering specific questions in micro-cultures that exist within larger cultures. ^11^ Given our study’s primary aim to understand feasibility of implementation, we employed our qualitative methods using an implementation science perspective.^12^

*Sample and Data Sources*

Participants were employees of Site 1, and were, one month prior to the site visit, notified via email from clinic leadership that a researcher would be visiting. Four sources of data were collected: observations, interviews, a focus group, and artifacts. Participants were allowed to partake in as many or few data collection activities as schedules allowed. Some participants took part in all activities, whereas others only took part in one activity. All participants provided oral consent for participation in the study. Ethics approval and oversight were provided by the Mayo Clinic IRB.

*Data Collection*

The data collection period took place over three days on-site (January 2 – 4, 2018), as well as a number of preparatory phone meetings and email exchanges. Fourteen people were invited to participate in interviews by email based on their high level of involvement in the *Capacity Coaching* program and role diversity; ultimately ten chose to participate including current and past directors of the Women’s Health Clinic, a social worker, a program manager, a nurse, a pharmacist, and a women’s health fellow. We also conducted a focus group with the current Women’s Health Clinic team and nine hours of observation. Some interviewees also participated in the focus group; the focus group was also enriched by team members who had more minor roles in the carry-out of the program. All data was collected by lead author (K.B.).

Observations occurred during clinic on- and off- hours; observations during clinic hours included patient interactions with health professionals and health professionals with each other. No patient protected health information was collected. Those observed were members of the PACT teams that participated in the pilot. Observations were conducted in the Women’s Health Clinic, in the mental health clinic, and in another primary care clinic where pharmacy consultation services were provided. Observations were conducted in areas beyond the Women’s Health Clinic, the primary pilot location, because all Women’s Health Clinic staff are not located in the same area of the center. Detailed observation notes were collected in the field, and at the end of each day, further written notes summarizing data collection activities were completed.

Focus group data was collected using a semi-structured interview guide informed by the NPT from the team perspective. ^8^ Individual interview data was collected using an unstructured interview format, beginning with a “grand tour” of the reason for the interview: to fully understand the experience of implementing *Capacity Coaching* in their practice.^13^ After letting the participants talk as long as they liked, follow-up questions probed at experience, such as asking about the influence of the program on their practice following the pilot period, detailed information about changes in day-to-day work, and sensitive group dynamic information, which was unlikely to emerge in the focus group discussion. Guides are included in **Appendix 2**. All focus groups and interviews were audio recorded and were transcribed verbatim. Artifacts provide by the team to K.B. included the final version of the implementation toolkit, workbook materials for coach-patient interactions, and template tools for documenting *Capacity Coaching* in the medical record. The implementation toolkit contained patient stories told in the first-person, as recreated by staff who delivered the intervention. We did not have IRB approval to interview patients for this study, and therefore, patient quotes were derived exclusively from the toolkit artifact.

*Reflexivity*

The practice of reflexivity “involves being deliberately aware of oneself, one’s responses, and one’s internal state in relation to a specific situation.”^11^ K.B. kept a reflexivity journal during time onsite and during the analysis period, tracking initial impressions, state of mind, and thoughts about ideas that need to be subjected to follow-up interview questions, observations, and discernment.

*Data Analysis*

All data sources including transcripts, artifacts, and observation notes, hereafter referred to as “source documents” were imported into Nvivo 11 for analysis. The analysis process used procedures guided by Roper and Shapira’s process for ethnographic analysis.^11^

First, the lead author (K.B.) and two trained research assistants (P.O., A.T.) listened to the audio recorded data and read all source documents, then coded source documents inductively using line-by-line coding to develop a code book. We used this process on three source documents, meeting two to three times weekly to discuss newly emerging codes and codes that should be combined or reconciled. We deemed the codebook complete after coding the third source document, as no new codes were emerging. We continued to meet twice weekly to address discrepancies or potentially new codes as we coded remaining source documents using the codebook; two new codes emerged from the later data and were added to the codebook. In addition to our inductive codes we included *a priori* deductive codes related to NPT: coherence, cognitive participation, collective action, and reflexive monitoring.

After coding, K.B. began to aggregating codes into themes using grouping and matrix functions in Nvivo 11. Coders reviewed results from the analysis to ensure that interpretations of the data remained close to the data. K.B. summarized all data into key themes found in the culture of the clinic, using CuCoM, NPT, and TPC. This step represents the use of well-fitting existing conceptual models and theories to organize the data after initial analyses in a manner which is useful for the translation of interventions into practice and is respected as particularly useful for meeting challenges in implementation of complex interventions.^12,14^

**Results**

*Summary of Capacity Coaching Intervention Delivered*

A core group of individuals championed the *Capacity Coaching* intervention, including a primary care physician, social worker, and PACT team nurse. The social worker served as the primary project coordinator, and a research coordinator kept up with data elements used to generate the implementation toolkit. These individuals met regularly and other clinicians and health professionals met with them periodically, particularly in the planning phases. The planning phase lasted approximately a year and the pilot implementation lasted six months (April 1, 2017 - October 31, 2017).

At the time of the workshop, Site 1 had planned to have the social worker serve as the Capacity Coach and Site 2 had a PSS as the Capacity Coach. During planning and the early pilot, it became apparent that Site 1 could also be best served by a PSS as the Capacity Coach, because patients placed value on the having shared lived experience as a female veteran living with chronic illness. Therefore, the PSS was brought on board by the team for Site 1. The Social Worker then trained the PSS and supervised her throughout the intervention period.

21 patients went through the program during the six-month implementation at Site 1 and patients interacted with the coach between 3 and 6 times, depending on their needs. This constituted a total of 21 in-person encounters and 42 telephone encounters. At the conclusion of the pilot, the team developed a freely-available *Capacity Coaching* toolkit to help other VA’s implement the program (available through VA Pulse or minimallydisruptivemedicine.org.) Core components of the intervention delivered appear in **Table 1**.

*Alignment with Cumulative Complexity Model*

There was evidence that the pilot *Capacity Coaching* program was aligned with the CuCoM. Specifically, there was considerable evidence that those most involved in the pilot focused on reducing patient work. For patients, the primary work added by the program was engaging with the coach. However, there was evidence that even this work was carefully engineered to be minimized, using strategies such as video cameras and iPads, patient-coach visits off-site, and warm hand-offs to the coach so that the first visit could occur immediately.

While formal outcomes were not collected from the program’s implementation, interviews and artifacts reflected success stories in patients’ ability access and use healthcare and enact self-care, as well as their outcomes and quality of life*.* Despite staff members’ description of how implementing *Capacity Coaching* impacted their practice (e.g., how they consider MDM in their everyday interactions with patients), there was no clear evidence from ethnographic observations that the program’s impacted to elsewhere in the center.

*Alignment with Theory of Patient Capacity*

Participant’s stories and artifacts illustrated that the implementation of the *Capacity Coaching* program positively acted upon each construct of the Theory of Patient Capacity (**Table 2)**.

*Implementation Successes and Challenges*

Beyond the impact of the program and its alignment with the principles of MDM, the remainder of the data was primarily focused on stories that were illustrative of the success and the challenges of implementation. These can be broken down into the four constructs of NPT: coherence, cognitive participation, collective action, and reflective monitoring (**Table 3)**. The primary success of the implementation was seen in the domains of coherence and collective action and challenges occurred in cognitive participation and reflexive monitoring.

*Transferability of Capacity Coaching Curriculum*

A surprising finding that emerged outside of the conceptual models and theories was the applicability of the *Capacity Coaching* curriculum beyond patients living with chronic conditions. One participant highlighted that she took the skills from the workshop and applied it to working with nursing students.

**Discussion**

Key findings are summarized in **Table 4**.

*Limitations and Strengths*

This evaluation was conducted after the pilot concluded and no continued *Capacity Coaching* activities occurred during the time the data was collected. This means the robustness of the evaluation relies on participants’ memories of the previous two years and may only capture key highlights of what occurred. Second, the evaluation was entirely qualitative, which means the impact of the program on patients’ health outcomes and quality of life relies entirely on anecdotal cases documented by staff. This limitation exists because of the narrow scope of the grant funding for the program, as well as the heterogeneous nature of the small patient population that participated in the pilot.

*Implications for Research and Practice*

Importantly, even in this brief pilot of the *Capacity Coaching* program, it satisfied the components of MDM, and addressed key needs of patients living with multimorbidity not addressed by other recent chronic care interventions, such as being agnostic to the chronic condition(s), acknowledging and reducing patient work, and supporting patients’ capacity holistically.^15^ This suggests *Capacity Coaching* may deserve broader testing to understand its impact on patient health outcomes, quality of life, and healthcare utilization.

Positive changes in a variety of outcomes have been elusive when testing team-based management interventions for multimorbidity.^16^ In studies, multiple interventions saw no effect on measures of utilization, health outcomes, or caregiver- or patient-reported outcomes. ^17-19^ One of these interventions was even specifically implemented within VA PACT teams.^19^ However, participants exposed to the *Capacity Coaching* program could clearly articulate the difference between it and other programs at the VA, including one for intensive management of patients with chronic conditions and numerous programs offered through Mental Health. This distinguishability suggests it should not be immediately lumped with past programs, but rather compared. Importantly, the fidelity of the program’s adherence to MDM principles should be monitored closely in future research, as it appears to be a distinguishing factor.

The HWC literature has lacked clarity in defining coaching.^20^ However, a recent systematic review of HWC in chronic conditions indicated statistically significant changes in patients psychological, behavioral, physiological, and social outcomes across 11 of 13 studies examined.^21^ Furthermore, a recent compendium of HWC interventions indicated mostly positive results for patients living with cancer, diabetes, heart disease, hypertension, and obesity.^22^ Finally, in a recent systematic review of HWC for cancer survivors, patient capacity was supported across four of the five constructs of the TPC.^23^ These previous studies also point usefulness in fully testing *Capacity Coaching*.

It is important that future research of *Capacity Coaching* and MDM-driven interventions incorporate the implementation learnings of this study. First, implementation was a complex process of integrating a new way of working into the primary care team, which filled much of the 6-month pilot period. There was little time to enroll others and to plan expansion or evaluation. Second, critical challenges in the cognitive participation of the full primary care team could potentially have been better addressed with a longer-term cultural change approach. Curriculum to consider includes Leadership Saves Lives (LSL), which was previously applied to address the problem of post-heart attack mortality. ^24^ In testing LSL, researchers found significant changes in culture across hospitals and decreased post-heart attack mortality rates in hospitals that demonstrated the greatest changes in their culture. ^25^ Therefore, there is potential to use LSL as a facilitation strategy to overcome implementation challenges faced when piloting *Capacity Coaching* to test its impact on patient health outcomes and quality of life*.*

**Conclusion**

This study represents the first implementation and evaluation *Capacity Coaching*. It demonstrated that the program was feasible to implement in team-based primary care and adhered to principles of MDM. We saw significant success of the implementation in participants making sense of the intervention (coherence) and working collectively to enact the program in the pilot period (collective action). Implementation challenges occurred in planning the work of implementation and enrolling a diverse coalition of clinical staff to expand referrals to the program (cognitive participation), and in evaluating the impact of the program on outcomes that upper leadership was interested in to continue the program beyond the grant funding period (reflexive monitoring). These challenges suggest the potential positive impact of incorporating existing culture-change curriculum, such as LSL, to facilitate implementation.

**Bibliography**

1. Barnett K, Mercer SW, Norbury M, Watt G, Wyke S, Guthrie B. Epidemiology of multimorbidity and implications for health care, research, and medical education: a cross-sectional study. *The Lancet.* 2012;380(9836):37-43.

2. May C, Montori VM, Mair FS. We need minimally disruptive medicine. *BMJ.* 2009;339:b2803.

3. Shippee ND, Shah ND, May CR, Mair FS, Montori VM. Cumulative complexity: a functional, patient-centered model of patient complexity can improve research and practice. *Journal of clinical epidemiology.* 2012;65(10):1041-1051.

4. Gallacher K, May CR, Montori VM, Mair FS. Understanding patients' experiences of treatment burden in chronic heart failure using normalization process theory. *Annals of family medicine.* 2011;9(3):235-243.

5. Gallacher K, Morrison D, Jani B, et al. Uncovering treatment burden as a key concept for stroke care: a systematic review of qualitative research. *PLoS medicine.* 2013;10(6):e1001473.

6. Shippee ND, Shippee TP, Hess EP, Beebe TJ. An observational study of emergency department utilization among enrollees of Minnesota Health Care Programs: financial and non-financial barriers have different associations. *BMC health services research.* 2014;14:62.

7. Boehmer KR, Gionfriddo MR, Rodriguez-Gutierrez R, et al. Patient capacity and constraints in the experience of chronic disease: a qualitative systematic review and thematic synthesis. *BMC family practice.* 2016;17(1):127.

8. May C, Murray, E., Finch, T., Mair, F., Treweek, S., Ballini, L., Macfarlane, A. and Rapley, T. Normalization Process Theory On-line Users’ Manual and Toolkit. <http://www.normalizationprocess.org>. Published 2010. Accessed January 13, 2014.

9. Boehmer KR, Guerton NM, Soyring J, Hargraves I, Dick S, Montori VM. Capacity Coaching: A New Strategy for Coaching Patients Living With Multimorbidity and Organizing Their Care. *Mayo Clin Proc.* 2019;94(2):278-286.

10. Boehmer KR, Hargraves IG, Allen SV, Matthews MR, Maher C, Montori VM. Meaningful Conversations in Living with and Treating Chronic Conditions: Development of the ICAN Discussion Aid. *BMC health services research.* 2016.

11. Roper JM, Shapira J. *Ethnography in nursing research.* Vol 1: Sage; 2000.

12. US Department of Health and Human Services. Qualitative Methods in Implementation Science. *National Institutes of Health Bethesda, MD, USA: National Cancer Institute.* 2018.

13. Rubin HJ, Rubin IS. *Qualitative interviewing: The art of hearing data.* Sage; 2011.

14. Sandelowski M. Theory unmasked: The uses and guises of theory in qualitative research. *Research in nursing & health.* 1993;16(3):213-218.

15. Boehmer KR, Dabrh AMA, Gionfriddo MR, Erwin P, Montori VM. Does the chronic care model meet the emerging needs of people living with multimorbidity? A systematic review and thematic synthesis. *PloS one.* 2018;13(2):e0190852.

16. Smith SM, Wallace E, O'Dowd T, Fortin M. Interventions for improving outcomes in patients with multimorbidity in primary care and community settings. *Cochrane Database Syst Rev.* 2016;3:CD006560.

17. Boult C, Reider L, Leff B, et al. The effect of guided care teams on the use of health services: results from a cluster-randomized controlled trial. *Archives of internal medicine.* 2011;171(5):460-466.

18. Hogg W, Lemelin J, Dahrouge S, et al. Randomized controlled trial of anticipatory and preventive multidisciplinary team care for complex patients in a community-based primary care setting. *Canadian family physician.* 2009;55(12):e76-e85.

19. Zulman DM, Chee CP, Ezeji-Okoye SC, et al. Effect of an Intensive Outpatient Program to Augment Primary Care for High-Need Veterans Affairs Patients: A Randomized Clinical Trial. *JAMA Internal Medicine.* 2016.

20. Boehmer KR, Barakat S, Ahn S, Prokop LJ, Erwin PJ, Murad MH. Health coaching interventions for persons with chronic conditions: a systematic review and meta-analysis protocol. *Systematic reviews.* 2016;5(1):146.

21. Kivelä K, Elo S, Kyngäs H, Kääriäinen M. The effects of health coaching on adult patients with chronic diseases: a systematic review. *Patient Education and Counseling.* 2014;97(2):147-157.

22. Sforzo GA, Kaye MP, Todorova I, et al. Compendium of the Health and Wellness Coaching Literature. *American Journal of Lifestyle Medicine.* 2017:1559827617708562.

23. Barakat S, Boehmer K, Abdelrahim M, et al. Does Health Coaching Grow Capacity in Cancer Survivors? A Systematic Review. *Population health management.* 2018;21(1):63-81.

24. Curry LA, Linnander EL, Brewster AL, Ting H, Krumholz HM, Bradley EH. Organizational culture change in U.S. hospitals: a mixed methods longitudinal intervention study. *Implementation science : IS.* 2015;10:29.

25. Curry LA, Brault MA, Linnander E, et al. Influencing organizational culture to improve hospital performance: A mixed methods intervention study. *Under Review.* 2017.

Figure 1: Orientation of Minimally Disruptive Medicine's Conceptual Frameworks and Middle-Range Theories

| **Table 1**: Core Components of Capacity Coaching Intervention Delivered | |
| --- | --- |
| **1** | Full healthcare team trained in Capacity Coaching with the autonomy to refer patients to the Capacity Coaching Program (e.g., a social worker could refer just as easily as a primary care clinician) |
| **2** | A peer mentor (PSS) with shared lived experience, prepared to meet patients where they were at rather than prescribe any new interventions for the patient |
| **3** | Materials and communication with patients to ensure they understood this was not an attempt to label them as “difficult patients” but rather help them with difficult life and healthcare situations |
| **4** | Capacity Coach knowledgeable in the available resources of the patients’ healthcare team and healthcare system broadly |

| **Table 2: Program Alignment with Theory of Patient Capacity** | | |
| --- | --- | --- |
| **Construct** | **Positive Impact** | **Representative Quotes** |
| Biography | While the capacity coach was originally the team’s social worker, they eventually transferred the coach role to a peer female-veteran.  The program allowed patients to express their stories, the changes in their lives because of their conditions, and work through how to discuss those with their doctors. | *“And that was another reason we thought [peer] would be a good match, because she’s a mother; she had a lot of face validity. She wasn’t just like a single female talking to somebody that is a caregiver and is juggling a zillion and one things. I mean she’s a veteran. She’s had those challenges and she’s a mom.” – Staff Member 1, clinician*  *“Back in the day, I used to play all sorts of sports: Field hockey, soccer, volleyball, you name it, I’d try it. I actually liked boot camp: I loved challenging my body like that. On my second tour to Iraq, the jeep I was in was overturned by an IED [improvised explosive device]. Broke both my legs. Healing and rehab took forever. I got really depressed and even when I was good enough to walk and do stuff again, I just didn’t want to. I gained a lot of weight and have zero motivation to do anything about it. About a year ago, I was diagnosed with diabetes. I’m ashamed to see my doctor. She didn’t know me when I was healthy; all she’s gonna see is someone who’s fat and lazy and not managing her diabetes very well. A friend of mine suggested I see this gal at the VA, a capacity coach. She told me about an online support group for women that helps them advocate for themselves with providers. Now I rehearse what I’m going to say and how I’m going to say it before I see my doctor. It’s helped me a lot. – Toolkit, Patient Success Story* |
| Resources | The capacity coach and social worker worked collaboratively, with the social worker supervising the coach and the two meeting weekly to discuss cases. This pairing worked well, and they were able to connect patients to resources in the VA or their community | *“There was one person who she broke her leg and she needed a wheelchair and I was like ‘Okay. Hmm. I can come and help a little bit’… And they were able to get the things that they needed and they were like ‘oh, that was so helpful.’ I recommended, you know, going to a senior center to that same person, and her partner really was appreciative of the things that I was coming to them with.” – Staff Member 2, Capacity Coach*  *“So she was with us probably five hours a week on Monday and Tuesday mornings. And we would touch base if not both days, um, one day. And we could go through her cases and updates, and I could say ‘Hey, that person would be great for OT [occupational therapy] lifestyle coaching’ or ‘Hey, let’s connect that person to the pharmacist.’” – Staff Member 4, Social Worker* |
| Environment | The program shifted the way in which the healthcare team was interacting with patients, as well as the way they worked together as a team to support patients. | *“My capacity coach gave me this journal with some stickers. I’ve been using it to track my moods, sleep, food cravings…stuff like that. I write my blood sugar readings in it, too. It’s helped me figure out some stuff out, make some connections. When I went to the doctor last week, my A1c was much better. My doctor was so happy! She asked if she could tell the team, and I said sure, and then everyone applauded, right there in the clinic! It was really nice to be recognized like this, to feel that positivity.” –Toolkit, Patient Success Story*  *“When I first met her, my first encounter with her, she had just found out that she had breast cancer. … She went inpatient in August. Because she had a reaction to the chemo, and it was a serious reaction. She was in palliative care – from August to December she was in there, and I would go and visit her, and ‘how you doin’?’ You know, and she would say - sometimes you know, uh, sometimes she might not have been in the best moods, but that is understandable. But other times I would go in, I’d visit her and she would say ‘You know what? I’m so glad that you came today. I’m gonna get out of the bed now. I feel better. I’m gonna walk around. I’m gonna take a shower.’” – Staff Member 2, Capacity Coach* |
| Work | The Capacity Coach was able to work with patients towards setting small, achievable goals that were in line with their values, preferences, and context. | *“[Peer] was awesome at doing goal setting and following up with them every week and meeting with them every week or every other week so she would do that, and she could leave the clinic which is really nice.” - Staff member 4, Social Worker*  *“My Capacity Coach told me about this phone app that reminds me to drink water, get up and stretch every 20 minutes, and park farther from my destination: It’s really motivating!” – Toolkit, Patient Success Story* |
| Social | Some of the toughest challenges patients encountered in caring for their health that the staff highlighted was balancing self-care with caregiving for others. The Capacity Coach was often able to support them in working through this balance as well as working productively with their social network. | *“My sister, god bless her, is always needin’ my car for this, that, and everything. I don’t mind helping her out, but now I can’t get to the grocery store when I need to, so I just pop in at the convenience store on my block for stuff, and all they’ve got is junk food. My capacity coach is helping me work out some ways to talk nicely to my sister about getting my car back.” – Toolkit, Patient Success Story*  *“So just it seems like there’s always like some other outside influence. There was another lady who she had lots of medical issues and a spouse who was not doin’ what he needed to be doin’. And she was so worried about tryin’ to keep thinks intact that her care fell off and right now… and she’s in like a rehabilitation center.” –Staff Member 2, Capacity Coach discussing the biggest barriers she had to help patients overcome* |

| **Table 3: Implementation Success and Challenges** | | |
| --- | --- | --- |
| **Construct** | **Success** | **Challenge** |
| Coherence | - The workshop getting everyone on the same page initially. *“Um, so that was really important. And that’s why everybody came to your workshop. And so, everybody had the same, basic understanding.” –Staff Member 3, Clinical Champion* - Human-Centered Design and continuous iteration of the program until they felt they achieved success. “*Um, and once we switched over from [social worker] to [peer] as the capacity coach, [it changed] completely. And then [social worker] supervised [peer] but, um, yeah, she connected completely differently with our women.” – Staff Member 3, Clinical Champion* - Describing patients that might be a good fit for the program. *“PSS [Peer-Support Specialist] informs PACT members and supervisor about what type of patient might benefit from meeting with her and participating in HCD- [human-centered design] driven Capacity Coaching, such as no shows, patients with multiple chronic conditions, polypharmacy issues, and patients who were doing well until ‘life happened’ (e.g., experienced a crisis).” – Toolkit, description of appropriate patients* - The program’s distinguishability from other programs offered. *“I finally got it and said, you know, ‘ these are not difficult patients. These are women with difficult lives.’ … I think that labeling as difficult patients, people, you know, that fits into like a lot of our mental health patients who don’t take their medicines, so they’re definitely not taking their other meds. But there’s kind of a different category. … We were trying to reach out more to the women whose lives fell apart for a little bit.” –Staff Member 3, Clinical Champion* - Modifying existing structures (templates, supervision logs, etc.) to fit the new program. *“These are the templates we use that we created for all the peers. And how do you take that and then we can change the template to have the capacity coaching pieces in there, which was fine, but those were all things that just hadn’t been considered.” –Staff Member 1, Clinician* | - Conveying changes about the program to others. *“And when we started out, it was just for diabetics, and like, I didn’t know that they went into other stuff.” – Staff Member 9, Nurse* - Building validity of the peer as Capacity Coach. *“[Peer] was also [clinician]’s patient. So, I think that was a barrier and a uniqueness to it as well – like conflict of interest kinds of things maybe.” – Staff Member 4, Social Worker “Yeah I wasn’t gonna say anything. Yes, I think it was.” –Staff Member 11, clinician* |
| Cognitive Participation |  | - Getting people involved clinically and throughout Women’s Health. “*I don’t really know what she [social worker] did because I wasn’t involved. But then eventually, the idea came out- came down from somewhere to use the peer support person.” – Staff Member 8, Program Manager* - A select few individuals driving the program forward. *“Uh I think some people were more aware than others, and I think if we – when we would remind them, they’d say ‘Oh, yeah!’ but then it quickly dissipates. [Clinical Champion] and [Clinical Champion] were better about it.” – Staff Member 4, Social Worker* - Clarifying and creating a streamlined referral process from clinic to coach. *“Not the providers.” –Staff Member 12, Nurse “Really? I have never done it.” –Staff Member 13, Nurse* - The amount of time to get all the logistics worked out to implement the program. *“Cause the infrastructure wasn’t there. If we did six months now, it would look completely different because you would be comin’ out of the gate running. Because things needed to- we used to go back and we’ll say ‘Okay, let’s uh, strengthen this piece here and do somethin’ different here.” Staff Member 1, Clinician* - Balancing planning logistics and focusing on the big picture of the program’s intended impact. *“On the one hand, it’s nice just sort of be in the presence of people that are sort of big picture thinkers, but on the other hand, it’s like okay, at some point, we have to just, you know, decide and do something.” – Staff member 5, Project Coordinator* - Co-location, visibility, and marketing of the capacity coaching program. *“I think [peer] needed to be in the clinic or like have space so that- for me, a lot of things are out of sight out of mind.” –Staff Member, Social Worker “Yeah ‘cause it woulda been nice if you woulda – you know, if we would’ve – if you woulda walked in the nurses and say ‘hey, have you been doing this?” –Staff Member 12, Nurse* |
| Collective Action | - Appropriate patients were referred to the program. - Five hours Monday and Tuesday were dedicated for the capacity coach to be in the primary care clinic. - Individual’s practices with patients did change because of the intervention. *“I think it was just having a little bit more focus on, you know, ‘cause my practice I kinda of had to figure it out those barriers, I needed to focus on those, um, before we had the training. But like how to approach focusing on them, and being more approachable to my patients, the veterans, um, on how to you know, get that out of them. And be a little bit more nurturing so to speak about how we get to that point, and maybe even having a better structure of how we facilitate doing that.” –Staff Member 6, Pharmacist* - When the program transitioned the capacity coaching role from social worker to peer, the coach and social worker had a productive working relationship with each other and with patients. *“[Peer] is awesome at coaching and has, like, all of those skill sets. So she, I think, did a lot better job and really brought the program to life more than I could.” –Staff Member 4, Social Worker* - Capacity coaching notes were entered into the electronic medical record with a summary of the visit and next steps. These were signed by coach, social worker, and the referring clinician. - The Capacity Coach successfully used the workshop curriculum to work with patients. *“I made, uh, some really good connections through the capacity coaching. And I was a little saddened that it ended.” –Staff Member 2, Capacity Coach* - The implementation team put out a Capacity Coaching toolkit for other VA Medical Centers to use, and it will be shared with 31 other sites. *“I think there are 31 sites that have – that now have, um, peer support people in primary care. And none of the sites reached out, but I reached out to the directors of those programs. As it’s through the directors of those programs that allowed access to be able to put this on. It’s called TMS, and it’s a teaching program. They get credit for it to go through the webinars. So they’re extending that out. And I think he sad that they have five sites that they really wanted to pilot [capacity coaching] with.” Staff Member 3, Clinical Champion* - ICAN Implementation was straightforward. *“I like how it’s more conversational. ‘What’s on your mind today?’ Those three [questions] are really strong.” Staff Member 1, Clinician* | - The referrals to the program were primarily driven by a few champions of the program. *“I’m surprised we didn’t get more referrals is the other thing. Um, because I know how many people that I see whether they’re male or female could’ve been helped.” Staff Member 6, Pharmacist* - Limited flexibility of the Capacity Coach’s time due to the fact she was shared with another program. *“Her supervisor was really strict on, like, ‘You’re there Monday and Tuesday morning from like, 10 to 12’ kind of deal, but that doesn’t work. Like that is a really small window, so she was able to give herself some more flexibility, which I really appreciated, and she would kind of weave people into her other schedule.” –Staff Member 4, Social Worker* |
| Reflexive Monitoring | - Participants involved in championing the program found value in it, making it worth continuing. *“It definitely, um, helped with frequency of um, well, shorter intervals of follow up and just going a little bit more in details about those things that I don’t have the time to do and realistically, I don’t think anyone in the PACT team has time to do with the patients.” –Staff Member 10, Clinical Champion* - Participants highlighted that the male population might also have benefitted from the program. *“Well, because, I mean, we’re [women] 7% of the population here, right? 7%, I mean that is tiny. And it’s really easy to just have it sort of dismissed or forgotten about. But if you include men, um, men are more interested in being involved and working on it.” –Staff Member 8, Program Manager* | - Failure to build in robust evaluation into the pilot. *“And sometimes I’m kind of like, it’s so – I feel like it’s sort of untested, ‘cause we didn’t do the formal evaluation. And they’re all like ‘oh yeah, sure we’ll [other peer support specialists] do this.’ And I’m like ‘Okay,’ so there’s a little trepidation there. It’s like, well, I don’t really, you know, have any p-values or anything.” –Staff Member 7, Project Coordinator*   *“They could actually say ‘what was the system burden?’ would be the way to do it just like in our – in mental health they look at like how many people are comin’ in usin’ ED services gotten acute services. You probably – especially with a longer pilot – you could say how did [capacity coaching] save them money because this person now is actually using their meds and they’re not coming in and they’re not having a long hospital stay and those outcomes would be the way to sell it.” –Staff Member 1, Clinician*   - Failure by referring clinicians to check back in with patients on the value they found from the program. *“I think it would’ve been a good idea if I did ask some of those patients ‘well, how is that going?’ But I, as a provider, didn’t necessarily do that.” –Staff Member 10, Clinical Champion* - Lack of planning regarding the sustainability of the program beyond the grant funding period. |

| **Table 4:** Summary of Findings | |
| --- | --- |
| **1** | *Capacity Coaching* was feasible in clinical practice. |
| **2** | *Capacity Coaching’s* implementation achieved changes in clinical practice that were aligned with Minimally Disruptive Medicine. |
| **3** | The program’s implementation strengths were in participants making sense of the intervention (coherence) and working collectively to enact the program in the pilot period (collective action). |
| **4** | The program’s implementation challenges were in planning the work of implementation and enrolling a diverse coalition of clinical staff to expand referrals to the program (cognitive participation), and in evaluating the impact of the program on outcomes that upper leadership was interested in to continue the program beyond the grant funding period (reflexive monitoring). |
