# Supplementary material for: Capacity Coaching: A Focused Ethnographic Evaluation in Clinical Practice
Source: Mayo Clin Proc Innov Qual Outcomes. 2020 Feb 17;4(2):190–202. doi: 10.1016/j.mayocpiqo.2019.11.002 (PMC7140014; doi:10.1016/j.mayocpiqo.2019.11.002)
Supplement: Supplemental Appendix 2 [file mmc2.pdf]

Interview Guide

Thank you for agreeing to this interview. Really, the whole purpose of talking with you is to understand the experience of implementing Capacity Coaching in your practice over the previous six months.

Can you tell me about that experience?

Have the participant expand on the following aspects of their story as needed.

a. How did this experience change how you think about how you care for patients as a (pharmacist, physician, nurse, etc.)?

b. How did implementing this model change your everyday activities in practice?

c. What surprised you about implementing Capacity Coaching?

d. What frustrated you about implementing it?

e. Anything else you would like to share about this experience?

USE AS NEEDED:

Can you elaborate on that…

Tell me more about what you just said about….

Focus Group Questions

1. As team members, can someone start off by telling me in your own words what the Capacity Coaching Program was about?
2. From your perspective, what was the purpose of the program?
3. How is Capacity Coaching different from other programs you refer to?
4. What do you feel the program required of you? What was the footprint on your everyday activities?

5. What were some of the barriers you experienced in implementing the program? What about barriers in sustaining it?
6. What value did you see in implementing the program? What value would you see in continuing it?
7. Who do you see as the drivers of making this program happen during the pilot? What did their championing of the project look like?
8. What else would you like us to know about the program? What could contribute to its success in the future?
